# Feasibility of Fraction Collection in HPLC Systems with Evaporative Light Scattering Detector: Analysis of *Pectinatella magnifica*

**DOI:** 10.3390/molecules21111495

**Published:** 2016-11-08

**Authors:** Jiří Pazourek, Karel Šmejkal

**Affiliations:** 1 Department of Chemical Drugs, Faculty of Pharmacy, University of Veterinary and Pharmaceutical Sciences, Palackého tr. 1946/1, Brno 61242, Czech Republic; 2 Department of Natural Drugs, Faculty of Pharmacy, University of Veterinary and Pharmaceutical Sciences, Palackého tr. 1946/1, Brno 61242, Czech Republic; karel.mejkal@post.cz

**Keywords:** fraction collection, evaporative light scattering detector, hydrophilic interaction liquid chromatography, mixed-mode chromatography, *Pectinatella magnifica*

## Abstract

The use of a liquid chromatography (LC) splitter inserted between an HPLC column and an evaporative light scattering detector (ELSD) is described. This paper aims to show the feasibility of using the splitter in an HPLC-ELSD system to fractionate a model mixture of analytes, namely salicin (2-(hydroxymethyl)-phenyl-β-d-glucopyranoside) and glucose. The retention factors and efficiency of the separation were studied under various temperatures and water contents in the mobile phase in order to clarify the mechanism of polyols separation on a diol column under the conditions of hydrophilic liquid chromatography (HILIC). Finally, the system was applied to a biological sample (a lyophilized colony gel of *Pectinatella magnifica*), where the presence of fructose and glucose was confirmed.

## 1. Introduction

### 1.1. Preparative Chromatography

Increasing demand for plant-based medicines, pharmaceuticals, cosmetics and other products calls for suitable techniques that provide for a quick and easy determination of the authenticity of crude materials and the quality assurance of herbal products. A leading technique in this respect is preparative liquid chromatography, namely counter-current chromatography (CCC) or high-performance liquid chromatography (HPLC).

The collection of the fraction is a key aspect of preparative chromatography for obtaining milligram quantities of pure natural products or for a following purification of the products obtained [1]. For semi-preparative chromatography, the identification of compounds in the sample very often begins with fraction collection if a mass spectrometric detector is not available or difficult to use (e.g., in identifying isomers of saccharides). Fractionation of the bioactive compounds obtained from complex natural extracts is also an essential step in the de novo identification and assessment of bioactivity in natural product research; for instance, Challal et al. have recently reported a high-load method of medium-performance LC (MPLC) with ELSD and UV detectors monitoring the separation process in parallel [2].

Many recent papers have reported preparative chromatography using an ELSD for the analysis of products of natural origin. He et al. [3] have described the preparative isolation and purification of glycine-conjugated cholic acids from *Pulvis Fellis Suis* by high-speed CCC coupled with an ELSD. Liu et al. [4] have connected high-speed CCC and ELSD by flow injection and applied this system for preparative isolation and purification of ginkgolide compounds obtained from *Ginkgo biloba*. Alkaloids from *Nitraria sibirica* leaves have recently been isolated by pH-zone-refining counter-current chromatography and a new alkaloid “schobemine” identified; to determine purity, the authors used an ELSD [5]. Julianti et al. [6] have reported that a leaf extract of *Carica papaya* has undergone semi-preparative separation with HPLC-ELSD. Antiplasmodial activity related to alkaloids has been confirmed; flavonols were also isolated from the extract and purified. Rojas et al. [7] have quantified the dissolved organic matter in waters after solid-phase extraction by a CCC method with a sequentially connected UV detector and an ELSD.

Since an ELSD is, in principle, destructive, it must be the last detector hyphenated in a separation system. The analyte is nebulized into an aerosol, which practically disables fractionation (detection is performed in the gaseous phase). A typical laboratory semi-preparative HPLC system employs a UV detector to control the timing of fraction collection.

### 1.2. ELSD

An ELSD is suitable for analytes that lack UV-vis chromophores, and is an alternative to a refractive index detector (RID). A RID can be used only for isocratic elution, which represents a serious limitation for analysis of real biological samples with complex matrices. The ELSD was invented in 1966, but it became interesting for practical use only in 1978, when it was considered as a detector for modern HPLC. Detectors of evaporative light scattering, made by companies such as the Varex Corporation (Rockville, MD, USA), S.E.D.E.R.E (Alfortville, France), and Applied Chromatography Systems Limited (Macclesfield, UK), have been commercially available since the 1980s; the number of papers describing applications of ELSDs has grown enormously in recent years [8]. The application of ELSDs is very often connected with saccharide analysis in the HILIC mode.

Almeling and Holzgrabe [9] have studied the influence of the experimental parameters on the use of HPLC-ELSD for the quality control monitoring of drug substances, especially the effect of different flow rates of the scavenger (nebulizing) gas. Triacylglycerols in oils and infant formulas have been analyzed by HPLC with an ELSD [10]. Recently, Arslan et al. have described the optimization of parameters of an ELSD in a fully automated three-dimensional column-switching SPE-FIA-HPLC system used for lipid characterization [11].

ELSDs are known to exhibit a non-linear response, although some authors have reported linear calibration curves [7,12]. The calibration curve can be linearized by using single-point calibration [13], which has been adapted and applied successfully to the mutarotation monitoring of monosaccharides [14].

### 1.3. HILIC

Hydrophilic interaction liquid chromatography (HILIC) has become very popular for the separation and determination of polar compounds such as proteins, peptides, amino acids, nucleotides, and carbohydrates. Applications of HILIC began to increase especially after 2003, and many can now be found in the literature [15,16]. The term HILIC or “aqueous normal phase chromatography” should reflect the fact that, unlike RP-HPLC, the stationary phase is polar; moreover, the mobile phase contains an excess of organic solvent, typically more than 80% (*v*/*v*) of acetonitrile.

The theory of HILIC has been described by Alpert et al. [17], and the theoretical background has been discussed by other investigators [18,19]. Hemström and Irgum [20] have matched arguments from data in the literature, supporting a partitioning or adsorption mechanism of HILIC. Finding evidence for both the models, they agreed with Alpert et al. [17] that ‘‘most of the real HILIC separations are in essence multimodal.’’ This explains why the term “mixed-mode chromatography” is used as an alternative name for HILIC nowadays [21]. Multimodal retention mechanisms, including ion exchange, hydrogen bonding, and hydrophobic and hydrophilic interactions, have been discussed in the literature. Interestingly, in the HILIC mode on a silica stationary phase, both positive and negative slopes of van’t Hoff plots have been reported for the same analytes (polar glycine derivatives), which can be explained by the existence of type A and type B silica [22].

The mechanism of retention in HILIC has been studied for particular analytes [23,24,25] and several models of retention have been proposed. Jin et al. [24] have suggested a retention equation combining an adsorption model (where ln *k′* is proportional to ln *c_w_*) and a partitioning model (where ln *k′* is proportional to *c_w_*) in the form:

(1) $$\ln k\prime =a+b\times \ln c_{w}+c\times c_{w}$$

where *k′* is the retention factor; *a*, *b*, and *c* are constants, and *c_w_* is the water fraction (or concentration) in the mobile phase.

**Figure 1:**
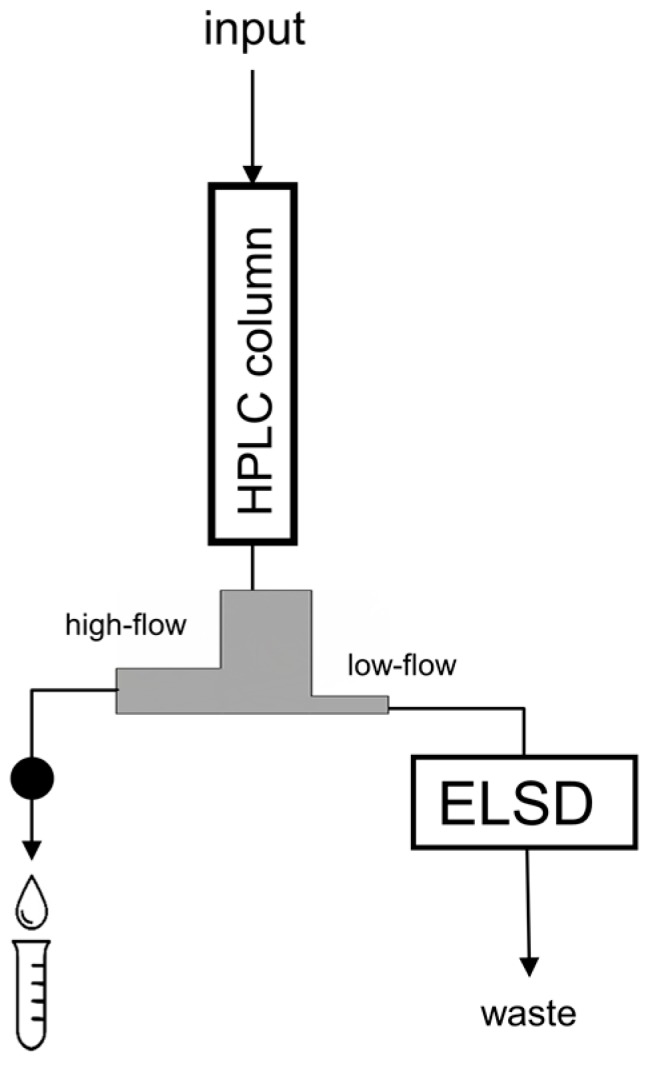
The set-up for collecting the fraction with an ELSD. The splitter is the grey segment connected below the HPLC column (high-flow and low-flow outlets are denoted); the black circle represents a UV detector monitoring salicin (for comparison purposes).

In this paper, we demonstrated that analytes with no suitable UV-chromophore could be collected for preparative purposes by using an ELSD. An LC-splitter (1:10) was inserted after the HPLC column (see Figure 1) with the low-flow outlet directed to an ELSD. The remaining (approximately 90%) sample could then be collected from the high-flow outlet of the splitter. Two model polar analytes were used, salicin and glucose, both of which give ELSD signals. Salicin also contains a UV-chromophore and can be detected simultaneously by using a UV detector. These model samples served first for a study of the separation mechanism and as a retention model of polyols on a diol column in HILIC mode. These analytes were then fractionated and re-injected, which proved the feasibility of the set-up. Finally, the method was applied to a biological sample, an extract from a lyophilized colony gel of the invertebrate *Pectinatella magnifica*.

## 2. Results and Discussion

In the first part of this section we compared the calibration curves of the model analytes (salicin and glucose). The experimental conditions used to obtain the calibration curves followed a paper of Pazourek [14] concerning the separation of monosaccharides (isocratic elution, mobile phase 10%:90% (*v*/*v*) water/acetonitrile, flow rate 2.0 mL/min), but the column temperature was changed to 25 °C. We then studied the separation mechanism of the HILIC using the retention of glucose at three different temperatures (10 °C, 25 °C, and 40 °C) and varying the elution strength of the mobile phase (3%, 5%, 10%, 15%, 20%, and 25% (*v*/*v*)), applying the retention model of Equation (1). In the last part of this section we demonstrated the performance of the LC-splitter in the HPLC system using the model analytes (salicin and glucose) and applied the set-up to a real sample of *P. magnifica*, which showed the presence of fructose and glucose.

### 2.1. Calibration Curves

**Figure 2:**
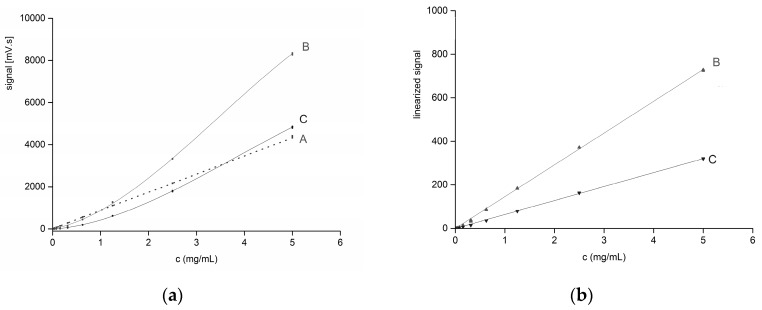
(**a**) Calibration curves of salicin (A, UV-detector λ = 270 nm, linear), and salicin with glucose (B, C, ELSD; non-linear); calibration points are connected by a cubic function only to visualize the course since there is no hard model for ELSD calibration functions; (**b**) Linearization of the ELSD calibration curves (B, C); peak areas were replaced by a function (area)x with exponents of x = 0.73 and 0.68, respectively [13]. Every concentration level was measured in triplicate. Experimental conditions: the mobile phase was 90% acetonitrile/10% water, flow rate was 2 mL/min, 25 °C, and injection volume was 10 µL.

Calibration curves were constructed for both the UV detector (270 nm, salicin) and the ELSD (salicin, glucose) in order to compare the detector signals and to observe the linearity of the curves. UV detection at 270 nm showed excellent linearity for the salicin calibration curve within the concentration range 0.02–5 mg/mL (Figure 2a, curve A; *R*^2^ = 0.9999). On the other hand, the ELSD signals for both salicin and glucose showed a typical upward concave course (Figure 2a, curves B and C) which can be clearly seen at the lowest concentrations (0.02–1 mg/mL) and is in agreement with previous observations on a similar DIOL column [14]. Single-point calibration linearization was used to make the ELSD calibration curves linear [13] (Figure 2b, curves B and C, exponents of 0.73 and 0.68, respectively).

A direct comparison of the sensitivities (i.e., the slopes of the calibration curves) of the UV detector and the ELSD calibration curves in Figure 2a is not worthwhile, because we did not select the optimum wavelength for UV detection. It is more reasonable to compare the signal-to-noise ratio (S/N), which is related to the limit of detection (LOD). In our case, at a level of 0.04 mg/mL, the S/N ratios were comparable for both detectors (S/N ≈ 40). The estimated LOD for the ELSD was 0.01 mg/mL (using successive dilution).

Although there are other analytical methods for the determination of glucose (some of them with higher sensitivity and/or with lower LODs—for review see [26]), we should note that the goal of this article is the demonstration of the fraction collection with ELSD. The analytes of glucose (and salicin) were chosen as model analytes.

### 2.2. Temperature Effect

The column temperature is an important parameter in an HPLC separation because it significantly affects the diffusivity of the analyte, the viscosity of the mobile phase, and the analyte transferring enthalpy between the stationary and mobile phase [22], especially when ionic interactions are involved [27]. In general, an increased temperature increases the diffusion coefficient and results in narrower peaks. At the same time, an elevated temperature could also result in a shorter retention time as explained by classical analyte diffusion theory. In the HILIC of monosaccharides, the column temperature can also affect the rate of conversion between α- and β-anomers of the analyte. Moreover, because the equilibration time of the process is typically about 1 h and the retention time is less than 10 min, the kinetics of mutarotation can easily be monitored with an ELSD [14].

The temperature effect on the separation process can be described by the van’t Hoff equation for the retention factor *k′*:

(2) $$\ln k\prime =-\frac{\Delta H}{\mathrm{RT}}+\frac{\Delta S}{R}+\ln\;\phi$$

where ΔH and ΔS are changes in enthalpy and entropy, respectively, between the mobile and stationary phases, R is the universal gas constant, and ϕ is the phase ratio.

**Figure 3:**
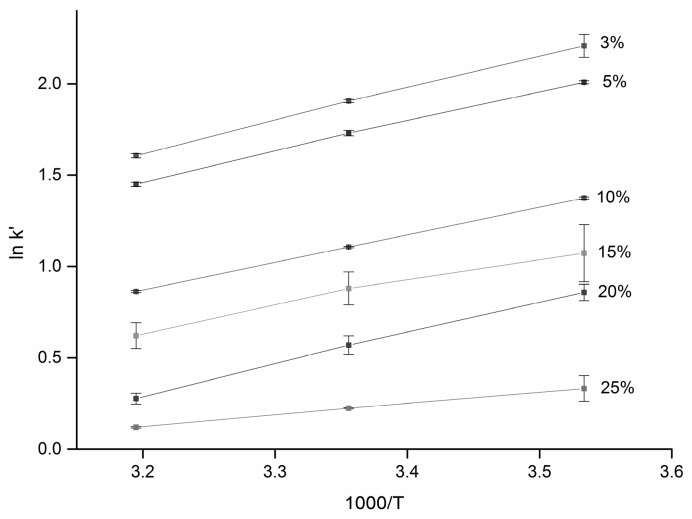
Thermodynamics of the separation—van’t Hoff plots. Retention factors were calculated for the second peak of glucose (β-anomer). Experimental conditions were as follows: flow rate 2 mL/min, injection volume 10 µL. The concentration range was 1–5 mg/L, at least three replicates at each of five concentration levels, all plots are linear within the measurement precision (error bars show the standard deviation of at least three replicates).

This equation should be followed if partitioning is involved in the separation mechanism. In order to elucidate the mechanism in our separation system of glucose on a diol stationary phase, calibration curves were recorded at temperatures 10 °C, 25 °C, and 40 °C. The corresponding average capacity factors of both the analytes were calculated, and van´t Hoff plots (ln *k′* vs. 1/T) for several different fractions of water in the mobile phase (3%–25% *v*/*v*) were constructed, as shown in Figure 3.

We observed quite good linearity of the van’t Hoff plots (*R*^2^ > 0.99) and positive slopes, which confirms that the transfer of the solutes from the mobile phase to the stationary phase is an exothermic process. Enthalpies were calculated for all of the compounds using the slope of Equation (2). For both salicin and glucose, the enthalpy of retention was negative, ranging from −14.75 kJ/mol (3% *v*/*v* of water) to −5.21 kJ/mol (25% *v*/*v* of water), although positive enthalpy changes have also been reported in HILIC [28]. Our results indicate that the transfer of the solutes from the mobile phase to the stationary phase is an exothermic process (favorable for low temperatures) and the solutes are retained more as the column temperature decreases. In accordance with this finding, the column plate number (N) for the retained peak of glucose (*k′* ≈ 4) doubled as the column temperature was reduced from 40 to 10 °C; on the other hand, N was practically constant for unretained peaks (*k′* ≈ 1 for salicin).

### 2.3. Effect of Water Concentration in the Mobile Phase

The commonly accepted model of HILIC presumes a stagnant layer of water on the hydrophilic surface of the stationary phase. Hydrophilic interactions including hydrogen bonding between the analytes and the stationary phase are therefore much more significant in HILIC separation than in RP-HPLC [22].

**Figure 4:**
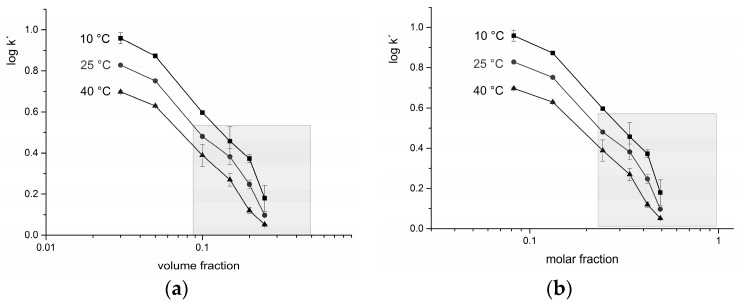
Elucidation of adsorption/partitioning mechanism of HILIC. Dependence of log *k′* on (**a**) volume fraction; and (**b**) molar fraction, respectively, of water in the mobile phase. The shaded area marks a low retention region that is not commonly applied; typical conditions for HILIC are up to 0.1 (volume fraction) or 0.2 (molar fraction) of water, respectively. Error bars show the standard deviations of at least three replicates.

A few recent studies have examined the relationship between the retention and the water content of the mobile phase in HILIC mode. These attempts have tried to determine whether the mechanism of retention is phase partitioning or surface adsorption or both [22,24,29,30]. From the theory of adsorption chromatography (Snyder-Soczewinski equation [20]) and from an empirical formula for neutral analytes partitioned between phases in RP-HPLC, it follows that in HILIC, a plot of log *k′* vs. log (mole fraction of water) should yield a straight line for an adsorption mechanism, whereas a plot of log *k′* vs. log (volume fraction of water) should yield a straight line for a partition mechanism [18]. Therefore, experiments were carried out varying the content of water in the mobile phase of 3%–25% (*v*/*v*). The results are shown in Figure 4.

Under the experimental conditions used for HILIC (5%–15% *v*/*v* of water in the mobile phase), neither graph showed a linear course even within the limits of the error bars, a fact which supports the theory of a mixed mechanism [21]. The shaded area in Figure 4 portrays conditions with low retention that are not used in practice.

**Figure 5:**
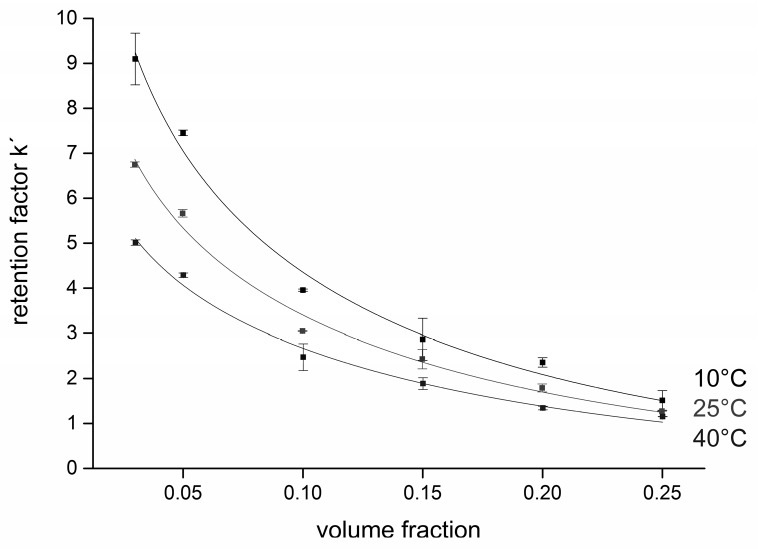
Verification of the retention model proposed by Jin et al. [24]. Experimental data were fitted with Equation (1) until the χ^2^ tolerance of 10^−6^ was reached (Levenberg-Marquardt algorithm). The values of the coefficients a, b, and c were as follows: +1.2, −0.3, −5.0 (upper curve, 10 °C); +1.0, −0.3, −5.0 (middle curve, 25 °C); +0.9, −0.3, −4.9 (lower curve, 40 °C). Error bars show the standard deviations of at least three replicates.

We also verified the retention model of the HILIC mode suggested by Jin et al. [24], who have claimed that their model of Equation (1) is more suitable than a purely mathematical fit (the quadratic polynomial ln *k′* = *c*0 + *c*1 × *c_w_* + *c*2 × *c_w_*^2^) because the coefficients *a*, *b*, and *c* of Equation (1) have a physico-chemical meaning: “*a* relates to the interaction energy between solutes with the stationary phase and the mobile phase, *b* relates to the direct analyte–stationary phase interaction, *c* relates to the interaction energy between solutes and solvents” [24]. Surprisingly, the authors put these constants forward only for the compounds of a plant extract separated on a β-cyclodextrin column (*a* ≈ −1, *b* ≈ −1, *c* ≈ −4, regression coefficients 0.998–0.999), and used the equation to predict the retention times. Our results are shown in Figure 5. A very good fit was found for each of the three temperatures tested; regression coefficients *R*^2^ were 0.9848 (10 °C), 0.9826 (25 °C) and 0.9851 (40 °C), respectively. The values of both the constants at 25 °C (*a* ≈ +1.0, *b* ≈ −0.3, and *c* ≈ −5.0, respectively) and the regression coefficients were comparable with those in the original paper [24].

### 2.4. Fractionation of a Model Mixture of Standards

**Figure 6:**
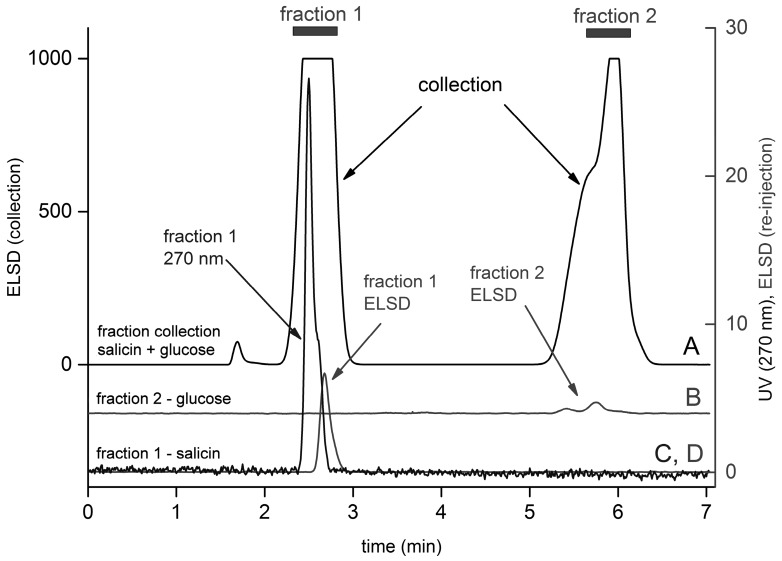
Fraction collection and re-injection of a model mixture. Chromatogram **A** is the ELSD signal used for fraction collection (fraction1 and fraction 2 were collected within the marked time intervals). Collection: 20 µL of a mixture of salicin and glucose (c = 10 mg/mL each) was injected. After re-injection, signals **C** (UV 270 nm) and **D** (ELSD) were obtained for salicin and chromatogram **B** for glucose (ELSD). Other experimental conditions: the temperature was 25 °C, the mobile phase was 90% acetonitrile/10% water, and the flow rate was 2 mL/min.

We demonstrated the performance of our HPLC system with the splitter on a model mixture of salicin and glucose standards. The results are shown in Figure 6. In order to obtain a sufficient signal from the re-injected fractions, a high amount of the analytes (20 µL, concentration 10 mg/mL, i.e., 0.02 mg) was loaded in the collection step (upper curve A, ELSD signal, left y-axis). Fractions were collected at around 2.6 min (salicin) and 5.8 min (glucose) (each fraction was collected for 30 s, i.e., 1 mL was collected). The fractions were then re-injected and the signals from both the detectors were recorded. To reduce the UV signal and make it comparable to the signal of the ELSD, a wavelength of 270 nm was chosen—curve C, right y-axis, fraction 1. Curves B and D (right y-axis) show the ELSD signals of fraction 1 and fraction 2, respectively; their retention times showed the presence of salicin in fraction 1 and glucose in fraction 2, respectively, as expected. Glucose exhibits the characteristic two-peak pattern representing its α- and β-anomers. The time delay between the peak maxima of curves C and D (salicin) reflects the time required for the analyte to travel from the UV detector to the ELSD (0.18 min at a flow rate of 2 mL/min, and 0.28 min at a flow rate of 1 mL/min, respectively).

### 2.5. Fractionation and Re-injection of an Extract from P. magnifica

The experimental set-up was used to identify the monosaccharides in a real sample, an extract taken from the inner colony gel of the invasive invertebrate *P. magnifica* [31]. The analysis was performed under the same conditions that we used for calibration (the mobile phase was 90% acetonitrile: 10% water (isocratic elution), the flow rate 2 mL/min, and the temperature 25 °C; the experimental parameters of the ELSD were as follows: the chamber temperature was 40 °C, and the nitrogen pressure in a standard nebulizer was 3.0 bars).

**Figure 7:**
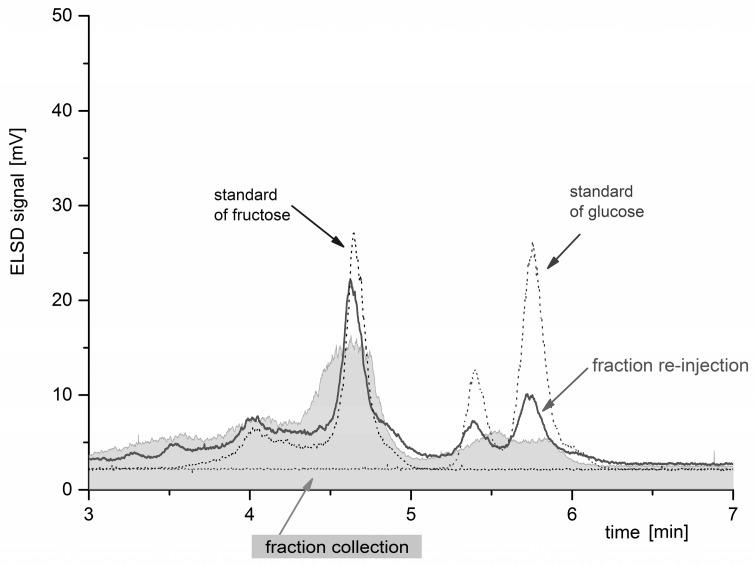
The splitter application to a real sample. The grey solid-filled chromatogram represents an extract of inner gel of *P. magnifica* used for fraction collection (injection volume was 50 μL, fractions were collected within 4–6 min). The fraction collected was evaporated to dryness under nitrogen, dissolved into 200 µL of water and then re-injected. The dotted traces show the chromatograms of the standards of fructose and glucose, respectively (each with a concentration of c = 2 mg/mL and an injection volume of 5 µL). Other experimental conditions: the temperature was 25 °C, the mobile phase was 90% acetonitrile/10% water, and the flow rate was 2 mL/min.

The results are shown in Figure 7: 50 µL of the *P. magnifica* extract was loaded on the column and, according to the ELSD signal, a fraction was collected between 4 min and 6 min (the grey-shaded chromatogram denoted “fraction collection”). The splitter was then removed, the fraction was evaporated to dryness under a nitrogen flow, dissolved into 200 µL of water, and re-injected (solid line chromatogram). The signals of both fructose and glucose were identified by their retention times and the typical anomeric patterns [14] (the dotted chromatograms of the fructose and glucose standards are overlapped).

## 3. Material and Methods

d-α-glucose, d-fructose, salicin, acetonitrile of HPLC grade and water of gradient HPLC grade were purchased from Sigma-Aldrich (St. Louis, MO, USA).

The stock solution of salicin and glucose (used for the study of the retention mechanism) was a mixture of standards, each with a concentration of 10 mg/mL. The individual stock solutions for the identification of glucose and fructose were also 10 mg/mL.

A YL9100 HPLC system (Young Lin, Anyang, Korea) connected to an ELSD (Agilent Technologies, Santa Clara, CA, USA) was used. The temperature of the ELSD chamber was set to 40 °C and the nitrogen pressure in the standard nebulizer was 3.0 bars. The column was LiChrospher100 DIOL (Merck, Darmstadt, Germany) 250 × 4.1 mm, with a packing particle diameter of 5 µm. Isocratic elution was always employed. If not stated otherwise, the mobile phase contained 10% (*v*/*v*) water and 90% (*v*/*v*) acetonitrile, the flow rate was 2.0 mL/min, the temperature of the column was 25 °C, and the injection volume was 10 µL.

### 3.1. Fractionation and Re-injection

For the fraction collection step, the splitter was inserted after the HPLC column (Figure 1), a high load of the sample was injected (20–50 µL), and the fraction was collected according to the ELSD signal. The splitter was then removed and the fraction(s) reinjected (10–50 µL). If the concentrations of the analytes were still low (e.g., for real samples), the fractions could be evaporated to dryness under flowing nitrogen and diluted in a suitable solvent prior to injection.

### 3.2. Sample from Pectinatella magnifica

*Pectinatella magnifica* is an invasive invertebrate that is spread world-wide and found in freshwater sources in the region of Třeboňsko (Třeboň, Czech Republic). Its biology, chemical composition, and environmental impact are not yet clear [31]. Because the composition of the animals (zooids) themselves is thought to be complex, only the inner gel of the colony blob, which represents most of the mass of the colony, was analyzed. The sample was collected in 2014.

After lyophilization, 100 mg of the material was weighed into 1.3 mL of a 1:1 water-methanol mixture [28], ultra-sonicated for 15 min, centrifuged for 10 min and then filtered (0.45 µm filter) into an HPLC vial. Fractions of the sample obtained from the splitter were evaporated to dryness under flowing nitrogen and dissolved in 200 µL of water prior to re-injection.

## 4. Conclusions

It was demonstrated that an LC-splitter inserted between an HPLC column and an ELSD can be adapted for fractionation and the following identification of compounds without a suitable UV-chromophore. Typical analytes for applications of such a chromatographic system are saccharides, or glycosides (common compounds in phytochemistry), and other polar compounds.

Analysis of a real sample of *P. magnifica* showed that the colony gel contains fructose and glucose, a fact which had not previously been reported. This finding is also in agreement with our tentative hypothesis (deduced from elemental analysis results) that the gel produced by *P. magnifica* is a highly glycosylated protein.

With respect to the separation mechanism of glucose on a diol column, van’t Hoff plots revealed that the transfer of the solutes from the mobile phase to the stationary phase is an exothermic process. Experiments varying the composition of the mobile phase to provide different elution strengths showed that neither adsorption nor partitioning is the prevailing mechanism; they rather suggested a mixed-mode (multimodal) mechanism of retention. The HILIC retention model of Equation (1) put forward by Jin et al. [24] predicts the retention factor k´ as a function of the water concentration in the mobile phase and is a suitable description of the mechanism of retention.

## References

[1] Hostettmann K., Marston A., Hostettmann M. (1998). Sample Preparation and Purification. Preparative Chromatography Techniques: Applications in Natural Product Isolation.

[2] Challal S., Queiroz E.F., Debrus B., Kloeti W., Guillarme D., Gupta M.P., Wolfender J.L. (2015). Rational and Efficient Preparative Isolation of Natural Products by MPLC-UV-ELSD based on HPLC to MPLC Gradient Transfer. Planta Med..

[3] He J., Li J., Sun W.J., Zhang T.Y., Ito Y. (2012). Preparative Isolation and Purification of Three Glycine-Conjugated Cholic Acids from *Pulvis Fellis Suis* by High-Speed Counter-Current Chromatography Coupled with ELSD Detection. J. Liq. Chromatogr. Relat. Technol..

[4] Liu J., Liu R.M., Sun A.L., Zhang Y.Q. (2013). Connection of High-speed Counter-Current Chromatography with Evaporative Light Scattering Detector by Flow Iinection and Its Application for Preparative Isolation and Purification of Gingkolide Compounds from Ginkgo Biloba L.. J. Liq. Chromatogr. Relat. Technol..

[5] Bakri M., Chen Q.B., Ma Q.L., Yang Y., Abdukadir A., Aisa H.A. (2015). Separation and purification of two new and two known alkaloids from leaves of *Nitraria sibirica* by pH-zone-refining counter-current chromatography. J. Chromatogr. B.

[6] Julianti T., De Mieri M., Zimmermann S., Ebrahimi S.N., Kaiser M., Neuburger M., Raith M., Brun R., Hamburger M. (2014). HPLC-based activity profiling for antiplasmodial compounds in the traditional Indonesian medicinal plant *Carica papaya* L. J. Ethnopharmacol..

[7] Rojas A., Sandron S., Wilson R., Davies N.W., Haddad P.R., Shellie R.A., Nesterenko P.N., Paull B. (2016). Simple, quantitative method for low molecular weight dissolved organic matter extracted from natural waters based upon high performance counter-current chromatography. Anal. Chim. Acta.

[8] Magnusson L.E., Risley D.S., Koropchak J.A. (2015). Aerosol-based detectors for liquid chromatography. J. Chromatogr. A.

[9] Almeling S., Holzgrabe U. (2010). Use of evaporative light scattering detection for the quality control of drug substances: Influence of different liquid chromatographic and evaporative light scattering detector parameters on the appearance of spike peaks. J. Chromatogr. A.

[10] Vyssotski M., Bloor S.J., Lagutin K., Wong H., Williams D.B.G. (2015). Efficient Separation and Analysis of Triacylglycerols: Quantitation of beta-Palmitate (OPO) in Oils and Infant Formulas. J. Agric. Food Chem..

[11] Arslan F.N., Kara H. (2016). Fully Automated Three-Dimensional Column-Switching SPE-FIA-HPLC System for the Characterization of Lipids by a Single Injection: Part I. Instrumental Design and Chemometric Approach to Assess the Effect of Experimental Settings on the Response of ELSD. J. Am. Oil Chem. Soc..

[12] Sun B.S., Gu L.J., Fang Z.M., Wang C.Y., Wang Z., Lee M.R., Li Z., Li J.J., Sung C.K. (2009). Simultaneous quantification of 19 ginsenosides in black ginseng developed from *Panax ginseng* by HPLC-ELSD. J. Pharm. Biomed..

[13] Kimball B.A., Arjo W.M., Johnston J.J. (2004). Single-Point Calibration with a Non-linear Detector: Carbohydrate Analysis of Conifer Needles by Hydrophobic Interaction Chromatography-Evaporative Light-Scattering Detection (HIC-ELSD). J. Liq. Chromatogr. Relat. Technol..

[14] Pazourek J. (2010). Monitoring of mutarotation of monosaccharides by hydrophilic interaction chromatography. J. Sep. Sci..

[15] Buszewski B., Noga S. (2012). Hydrophilic interaction liquid chromatography (HILIC)—A powerful separation technique. Anal. Bioanal. Chem..

[16] Groskreutz S.R., Stoll D.R. (2013). Theory and Practice of Two-Dimensional Liquid Chromatography Separations Involving the HILIC Mode of Separation. Hydrophilic Interaction Chromatography.

[17] Alpert A.J., Shukla M., Shukla A.K., Zieske L.R., Yuen S.W., Ferguson M.A.J., Mehlert A., Pauly M., Orlando R. (1994). Hydrophilic-interaction chromatography of complex carbohydrates. J. Chromatogr. A.

[18] McCalley D.V. (2010). Study of the selectivity, retention mechanisms and performance of alternative silica-based stationary phases for separation of ionised solutes in hydrophilic interaction chromatography. J. Chromatogr. A.

[19] Gritti F., Guiochon G. (2013). Mass transfer mechanism in hydrophilic interaction chromatography. J. Chromatogr. A.

[20] Hemström P., Irgum K. (2006). Hydrophilic interaction chromatography. J. Sep. Sci..

[21] Yang Y., Geng X. (2011). Mixed-mode chromatography and its applications to biopolymers. J. Chromatogr. A.

[22] Hao Z., Xiao B., Weng N. (2008). Impact of column temperature and mobile phase components on selectivity of hydrophilic interaction chromatography (HILIC). J. Sep. Sci..

[23] Guo Y., Gaiki S. (2011). Retention and selectivity of stationary phases for hydrophilic interaction chromatography. J. Chromatogr. A.

[24] Jin G.W., Guo Z.M., Zhang F.F., Xue X.Y., Jin Y., Liang X.M. (2008). Study on the retention equation in hydrophilic interaction liquid chromatography. Talanta.

[25] Karatapanis A.E., Fiamegos Y.C., Stalikas C.D. (2010). Study of the Behavior of Water-Soluble Vitamins in HILIC on a Diol Column. Chromatographia.

[26] Galant A.L., Kaufman R.C., Wilson J.D. (2015). Glucose: Detection and analysis. Food Chemistry.

[27] Dolan J.W. (2002). Temperature selectivity in reversed-phase high performance liquid chromatography. J. Chromatogr. A.

[28] Dong L., Huang J. (2007). Effect of Temperature on the Chromatographic Behavior of Epirubicin and its Analogues on High Purity Silica Using Reversed-Phase Solvents. Chromatographia.

[29] Liu M., Chen E.X., Ji R., Semin D. (2008). Stability-indicating hydrophilic interaction liquid chromatography method for highly polar and basic compounds. J. Chromatogr. A.

[30] Karatapanis A.E., Flamegos Y.C., Stalikas C.D. (2009). HILIC separation and quantitation of water-soluble vitamins using diol column. J. Sep. Sci..

[31] Balounová Z., Rajchard J., Švehla J., Šmahel L. (2011). The onset of invasion of bryozoan *Pectinatella magnifica* in South Bohemia (Czech Republic). Biologia.

